# Macrophage-driven inflammation in inflammatory bowel disease: mechanisms and therapeutic opportunities

**DOI:** 10.3389/fbioe.2026.1856903

**Published:** 2026-07-15

**Authors:** Marta Rossi, Alessandra Spanò, Apolline Fortun, Nikita Camilleri, Marta Magatti, Andrea Papait, Antonietta Silini, Ornella Parolini

**Affiliations:** 1 Department of Life Science and Public Health, Università Cattolica del Sacro Cuore, Rome, Italy; 2 Centro di Ricerca E. Menni, Fondazione Poliambulanza Istituto Ospedaliero, Brescia, Italy; 3 Fondazione Policlinico Universitario “Agostino Gemelli” IRCCS, Rome, Italy; 4 Fondazione IRCCS Casa Sollievo della Sofferenza, San Giovanni Rotondo, Italy

**Keywords:** Crohn’s disease, IBD, macrophages, trained immunity, ulcerative colitis, inflammasome

## Abstract

Gut inflammation can be triggered by multiple factors, including the loss of intestinal homeostasis and dysregulation of the innate immune system, which compromise the epithelial barrier and lead to tissue damage. Intestinal innate immunity protects the host from invading pathogens and limits microbial translocation while maintaining tolerance toward the commensal microbiota. Disruption of this balance is considered an important contributor to intestinal inflammation in inflammatory bowel diseases (IBD), encompassing ulcerative colitis (UC) and Crohn’s disease (CD). While IBD pathogenesis encompasses genetic susceptibility, epigenomic dysregulation, and environmental factors, this mini-review focuses on innate immune and macrophage-driven mechanisms that integrate these upstream signals into chronic mucosal inflammation. In this context, macrophages are key innate immune cells that provide a rapid first line of defense against conserved microbial and danger signals and play a central role in initiating and sustaining inflammatory responses. In this mini-review, we describe how disruption of intestinal homeostasis triggers activation of the innate immune system, including the recruitment and activation of macrophages. Specifically, we examine the functional polarization of macrophages during inflammation and its impact on disease progression in UC and CD. We highlight the role of inflammasomes, central components of innate immune signaling, which mediate the release of pro-inflammatory cytokines and pyroptotic cell death, thereby exacerbating tissue damage and disrupting host–microbiota interactions. We also discuss trained immunity, a process through which macrophages undergo long-lasting changes following repeated inflammatory signals, which may enhance their responses to future stimuli and contribute to persistent inflammation and disease recurrence in IBD. Finally, we review therapeutic strategies targeting macrophages and innate immune pathways. Despite clinical advances, current therapies remain limited and fail to address the complex inflammatory networks underlying IBD. A deeper understanding of innate immune and inflammasome-related pathways will be relevant for the development of multitargeted therapeutic strategies in IBD.

## Introduction

UC and CD are the two main forms of IBD, sharing dysregulated mucosal immune responses but differing in anatomical distribution, inflammatory depth, and molecular pathogenesis (Calvez et al., 2025), (Ramos and Papadakis, 2019). Genetic susceptibility and environmental triggers converge to drive chronic inflammation through aberrant mucosal immune activation in both conditions (Ramos and Papadakis, 2019), (Jostins et al., 2012). Although adaptive immunity contributes to disease pathogenesis through antigen-specific T and B cell responses, increasing evidence highlights innate immune dysfunction as an important contributor to mucosal inflammation (Chang, 2020), (de Souza and Fiocchi, 2016). Within the intestinal lamina propria, macrophages integrate microbial, epithelial, and stromal signals to orchestrate local immune responses (Zhang et al., 2023), (Chen et al., 2025).

**FIGURE 1:**
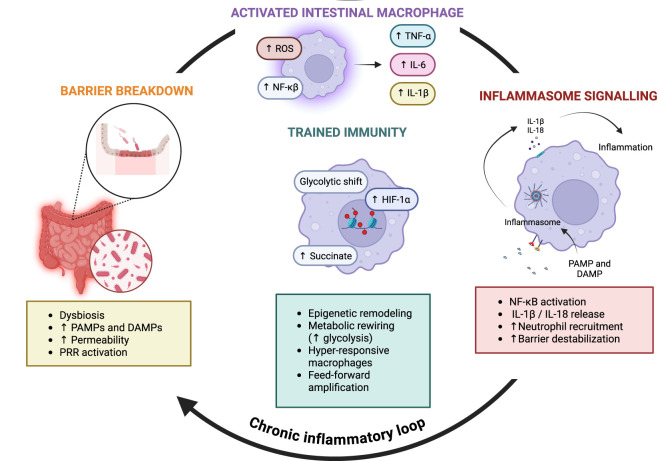
A self-sustaining macrophage inflammatory circuit in IBD. Barrier disruption and microbial translocation promote activation of intestinal macrophages through pattern recognition receptor signaling. Activated macrophages amplify inflammation via NF-κB–dependent cytokine production and NLRP3 inflammasome assembly, leading to caspase-1 activation and IL-1β maturation. Sustained inflammatory signals induce metabolic and epigenetic reprogramming consistent with trained immunity, characterized by increased glycolysis, HIF-1α stabilization and succinate accumulation. These changes reinforce pro-inflammatory gene expression and sustain cytokine production. The resulting feed-forward loop perpetuates epithelial damage and chronic mucosal inflammation in IBD. Created with Biorender.com.

Under physiological conditions, resident macrophages contribute to maintaining tolerance by sensing microbial signals while limiting excessive inflammatory responses through anti-inflammatory mediators, supporting regulatory T cell responses and performing silent phagocytosis, thereby preserving barrier integrity and supporting tissue repair (Zhang et al., 2023), (Chen et al., 2025). In IBD, epithelial barrier disruption facilitates microbial translocation and chemokine-driven circulating monocyte recruitment, leading to accumulation of inflammatory macrophages in the intestinal mucosa and impaired resolution of inflammation (Bain and Schridde, 2018), (Bain and Mowat, 2014), (Bain et al., 2014). Emerging mechanisms, including inflammasome activation and trained immunity, have expanded our understanding of how macrophage-driven innate circuits sustain chronic inflammatory processes, contributing to disease progression and, in some contexts, resistance to current therapies (Ochando et al., 2023), (Scalavino et al., 2024) (Figure 1).

## When homeostasis fails: macrophage activation in gut inflammation and mucosal barrier dysfunction

In IBD, disruption of intestinal homeostasis increases epithelial permeability and microbial translocation, exposing lamina propria immune cells to pathogen- (PAMPs) and damage-associated molecular patterns (DAMPs). These signals are sensed through pattern recognition receptors, including Toll-like and NOD-like receptors expressed by intestinal epithelial cells and macrophages (Lavelle and Sokol, 2020), (Rakoff-Nahoum et al., 2004), (Neurath, 2014). This early sensing phase represents a critical pathogenic switch: NF-κB activation induces IL-1β, IL-6, TNF, and IL-23, initiating inflammatory cascade that precedes adaptive immune responses (Neurath, 2014).

Within this circuit, macrophages act as key amplifiers of mucosal inflammation. Chemokines released during epithelial stress and early inflammatory responses promote recruitment of circulating monocytes to the intestinal mucosa, where they differentiate into cytokine-producing macrophages, a central feature of IBD pathology (Bain and Schridde, 2018), (Bain et al., 2014). Once activated, these cells reinforce epithelial dysfunction through TNF-α and IL-1β signaling, which disrupt tight junction organization by upregulating pore-forming proteins such as claudin-2 and reducing expression of occludin and zonula occludens-1, thereby promoting barrier leakage and microbial penetration (Lissner et al., 2015), (Horowitz et al., 2023), (Capaldo, 2023). In parallel, activation of the NLRP3 inflammasome integrates microbial and stress-related signals, leading to caspase-1 activation, maturation of IL-1β and IL-18, and pyroptosis (Zaki et al., 2010a), (Tourkochristou et al., 2019).

Importantly, sustained inflammatory exposure induces metabolic rewiring and epigenetic remodeling. Enhanced glycolysis and accumulation of metabolites, such as succinate, potentiate inflammatory gene expression and cytokine production (Arts et al., 2018). Stable chromatin remodeling at pro-inflammatory loci primes macrophages for exaggerated responses upon secondary stimulation, a process consistent with trained immunity (Saeed et al., 2014), (Netea et al., 2011). These mechanisms may uncouple inflammatory activation from its initial trigger, contributing to disease persistence and relapse.

Together, macrophage activation, inflammasome activation, and metabolic reprogramming establish a self-sustaining pathogenic circuit in IBD. While these mechanisms operate in both UC and CD, their relative weight differs: barrier dysfunction predominates in UC, whereas defective microbial clearance and transmural involvement characterize CD. Understanding how this circuit is initiated and stabilized provides the conceptual basis for therapeutic strategies aimed at reshaping macrophage plasticity and interrupting innate immune amplification (Figure 1).

## Macrophage recruitment and polarization as drivers of intestinal pathology

Under physiological conditions, lamina propria macrophages mainly derive from circulating monocytes and acquire a tolerogenic phenotype characterized by high phagocytic capacity and limited pro-inflammatory cytokine production (Chen et al., 2025), (Ma et al., 2025), (Saez et al., 2023). In IBD, epithelial barrier disruption and increased microbial translocation promote excessive monocyte recruitment through chemokines such as C–C motif chemokine ligand 2 (CCL2), C–C motif chemokine ligand 5 (CCL5) and C–X3–C motif chemokine ligand 1 (CX3CL1) produced within the inflamed mucosa. Epithelial cells and stromal fibroblasts act as major sources of CCL2 and CCL5, while CX3CL1 is produced by resident macrophages, dendritic cells and endothelial cells, leading to the accumulation of inflammatory macrophages in the intestinal tissue (Chen et al., 2025), (Lu et al., 2024).

Recruited monocytes differentiate into macrophages whose activation states are shaped by cytokines, microbial signals and stromal cues within the intestinal microenvironment, resulting in sustained immune activation (Saez et al., 2023), (Hegarty et al., 2023). Rather than a binary M1/M2 model, intestinal macrophages in IBD display a continuum of activation states, with individual cells co-expressing markers of both pro-inflammatory and regulatory phenotypes (Chen et al., 2025), (Ning et al., 2024), (Garrido-Trigo et al., 2023). At the pro-inflammatory end of this spectrum, interferon-γ (IFN-γ) and tumor necrosis factor-α (TNF-α) driven macrophages produce high levels of interleukin-1β (IL-1β), interleukin-6 (IL-6), TNF-α and reactive oxygen species (ROS), promoting epithelial injury and sustaining Th1/Th17-driven immune responses (Saez et al., 2023), (Lu et al., 2024). The regulatory subsets, in contrast, support tissue repair, immune regulation and angiogenesis through the secretion of anti-inflammatory mediators such as interleukin-10 (IL-10), transforming growth factor-β (TGF-β), and growth factors including VEGF and EGF, although their functions may become dysregulated in chronic inflammation (Ma et al., 2025), (Branco et al., 2024), (Magnusson et al., 2016).

High-resolution single-cell RNA sequencing (scRNA-seq), spatial transcriptomics and emerging multi-omic approaches have refined our understanding of macrophage heterogeneity in IBD. Within this framework, these technologies show that intestinal macrophages comprise multiple transcriptionally and spatially distinct populations organized within specialized tissue niches and reshaped during inflammation (Garrido-Trigo et al., 2023), (Domanska et al., 2022), (Mitsialis et al., 2020), (Liu et al., 2024). These include resident FOLR2^+^/C1QC^+^ macrophages, inflammatory IL1B^+^ macrophages, and inflammation-dependent alternative (IDA) macrophages distributed across mucosal niches (Garrido-Trigo et al., 2023), (Domanska et al., 2022), (Mitsialis et al., 2020). Multi-omics integration of scRNA-seq and chromatin accessibility suggest that enhancer remodeling contributes to macrophage-state stability and disease-specific transcriptional programs (Gudiño et al., 2025). These studies also reveal that macrophage heterogeneity differs systematically between UC and CD. In UC, inflammatory macrophage infiltration is predominantly confined to the mucosa and is associated with CCR2-dependent recruitment of circulating monocytes, impared differentiation into homeostatic resident macrophages and accumulation of pro-inflammatory monocyte-derived macrophages enriched for IL1B, TNF, CXCL8, and CD14 expression within active lesions (Mennillo et al., 2024), (Magnusson et al., 2015), (Smillie et al., 2019), (Li et al., 2026). By contrast, CD exhibits a broader and more heterogeneous macrophage compartment extending across the intestinal wall, including inflammatory, IDA and tissue-remodeling macrophage states that mirror its transmural pathology and chronic stromal activation (Garrido-Trigo et al., 2023), (Martin et al., 2019), (Chapuy et al., 2019). Critically, this disease-specific heterogeneity has direct clinical relevance, as inflammatory IL1B-expressing macrophages are enriched in phagocyte-rich lesions associated with anti-TNF resistance, whereas spatial analyses identify SPP1+ macrophage niches linked to fibroblast activation, epithelial injury, granuloma formation and persistent tissue remodelling (Martin et al., 2019), (Sanzo Machuca et al., 2026), (Gao et al., 2023), (Zhou P. et al., 2025), (Yang and Zhu, 2026).

Bidirectional interactions between macrophages and the intestinal barrier further exacerbate mucosal pathology. Inflammatory macrophages reinforce epithelial barrier dysfunction through cytokine-dependent pathways, thereby sustaining microbial translocation and chronic immune activation (Lissner et al., 2015), (Kaminsky et al., 2021). Conversely, under homeostatic conditions, microbiota-conditioned macrophages support barrier repair through the release of extracellular vesicles carrying pro-resolving signals that promote tight junction reassembly and epithelial restitution (Bain and Schridde, 2018), (Yang et al., 2019), (Díaz-Garrido et al., 2021). In the inflamed gut, this reparative capacity is lost, and danger signals released by damaged epithelial cells, including extracellular ATP and HMGB1, instead reinforce macrophage activation via NLRP3 and TLR4 signaling, establishing a tightly coupled feed-forward circuit between barrier disruption and macrophage activation (Zhang et al., 2025), (Zhu et al., 2025). In parallel, macrophages establish a wide cross-talk with the gut microbiota, sensing dysbiosis and shaping immune responses through pattern recognition receptors and metabolic pathways (Ning et al., 2024). Changes in microbial composition can directly affect macrophage differentiation and function, further promoting self-sustaining inflammatory circuits.

Recent evidence also highlights the importance of macrophage interactions with intestinal stem cells. Inflammatory macrophages inhibit stem cell renewal and differentiation, whereas reparative subsets support crypt maintenance and epithelial regeneration (Quan et al., 2025). Disruption of this macrophage–stem cell axis contributes to impaired mucosal healing and disease persistence.

These findings underscore that aberrant macrophage recruitment and polarization are major drivers of mucosal pathology in IBD and suggest that restoring macrophage functional balance through modulation of monocyte trafficking and inflammatory reprogramming represents a promising therapeutic strategy (Chen et al., 2025), (Lu et al., 2024), (Chang et al., 2014), (Na et al., 2019).

## Inflammasomes as central amplifiers of macrophage-driven innate immune signaling and tissue damage in the gut

Beyond macrophage recruitment and polarization, intracellular danger-sensing platforms drive the amplification of intestinal inflammation. Among these, inflammasomes are key molecular amplifiers of inflammatory signaling.

Inflammasomes are cytosolic multiprotein complexes that sense microbial invasion and cellular stress and couple innate immune recognition to downstream inflammatory effector pathways in the gut mucosa (Scalavino et al., 2024). These complexes regulate caspase-1 activation and the maturation of IL-1β and IL-18, central mediators of inflammation and pyroptotic cell death (Zaki et al., 2010a), (Tourkochristou et al., 2019). While controlled inflammasome activation maintains mucosal defense and homeostasis, its dysregulation contributes to chronic inflammation and tissue injury in IBD (Zhen and Zhang, 2019), (de Zoete et al., 2014).

Among inflammasomes, NOD-like receptor family pyrin domain-containing 3 (NLRP3) has been most extensively studied in IBD, functioning as a prominent integrator of danger signals such as extracellular adenosine triphosphate (ATP), ionic fluxes, mitochondrial dysfunction and microbial metabolites (Gong et al., 2025), (Boyapati et al., 2018), (Bernardazzi et al., 2022), (Macia et al., 2015). Elevated NLRP3 activation correlates with increased IL-1β and IL-18 production in inflamed intestinal tissues, which amplify leukocyte recruitment, promote Th17 responses and impair epithelial barrier integrity, thereby driving mucosal pathology (Tourkochristou et al., 2019), (Zhen and Zhang, 2019). However, accumulating evidence indicates that the role of NLRP3 in intestinal inflammation is complex. Distinct experimental models show both pathogenic and homeostatic outcomes depending on context, suggesting that balanced inflammasome activity is essential for intestinal health (Song et al., 2021), (Bauer et al., 2010). Notably, the contribution of NLRP3 differs across IBD subtypes. In UC, macrophage-driven activation of the NLRP3 inflammasome in the mucosa is strongly associated with IL-1β release, pyroptotic cell death and epithelial tissue injury, whereas in CD these pathways intersect with autophagy defects linked to ATG16L1 and NOD2 variants, resulting in impaired inflammasome regulation, persistent macrophage activation and chronic granulomatous inflammation (Liu et al., 2026), (Homer et al., 2010), (Gorreja et al., 2022). Macrophages are major contributors to inflammasome activation in the gut, while intestinal epithelial cells can also engage inflammasome signaling. Macrophages represent the primary source of inflammasome-derived cytokines, with NLRP3 integrating signals from pattern recognition receptors, metabolic stress, and microbial dysbiosis (Song et al., 2021), (Zaki et al., 2010b). IL-1β released from inflammasome-activated macrophages reinforces pro-inflammatory networks and drives further immune cell infiltration, while IL-18 modulates epithelial turnover and barrier function. Sustained overproduction of these cytokines favors the transition from protective immune responses to chronic inflammation. In addition to NLRP3, other inflammasomes such as NOD-like receptor family pyrin domain-containing 6 (NLRP6) and Absent in melanoma 2 (AIM2) contribute to microbial ecology and epithelial repair, highlighting the context-dependent functions of inflammasome complexes in intestinal homeostasis and disease (Scalavino et al., 2024), (Wlodarska et al., 2014).

Importantly enhanced inflammasome-driven pyroptosis amplifies tissue injury in IBD. Excessive cell death in epithelial and immune compartments compromises barrier integrity and releases damage-associated molecular patterns, creating a loop that perpetuates inflammation (Zaki et al., 2010a), (Zaki et al., 2011). Genetic studies also highlight that polymorphisms in inflammasome-related genes can influence disease susceptibility and progression, further linking dysregulated inflammasome signaling to IBD pathogenesis (Jostins et al., 2012), (Zhen and Zhang, 2019).

Because inflammasomes integrate upstream danger signals with downstream inflammatory responses, they represent promising therapeutic targets in IBD (Scalavino et al., 2024), (Zaki et al., 2011).

## Trained immunity and the persistence of inflammatory responses

In addition to chronic inflammasome activation, macrophages may acquire innate immune memory, also referred to as trained immunity, that sustains inflammation.

Traditionally, immunological memory was attributed exclusively to adaptive immune cells, enabling faster and stronger responses upon re-exposure to a pathogen. Trained immunity represents a functional memory in innate cells that challenges the classical dichotomy between innate and adaptive immunity, whereby prior exposure to microbial or stress signals induces enhanced responses upon subsequent stimuli (Ochando et al., 2023), (Netea et al., 2011). Unlike adaptive memory, trained immunity is mediated primarily by long-term epigenetic changes, such as histone modifications and DNA methylation, and metabolic rewiring (Saeed et al., 2014), (Fanucchi et al., 2021), (Quintin et al., 2012). While trained immunity likely evolved to enhance protection against infections, maladaptive innate training has increasingly been implicated in chronic inflammatory and autoimmune diseases, including IBD (Arts et al., 2018), (Netea et al., 2020).

In IBD, repeated exposure to PAMPs, DAMPs and microbial translocation due to epithelial barrier dysfunction creates an environment that promotes maladaptive immune training of tissue-resident macrophages (Robles-Vera et al., 2025), (Jentho and Weis, 2021). This process is characterized by stable epigenetic alterations, including enrichment of activating histone marks such as H3K4me3 and H3K27ac at promoters of pro-inflammatory genes, leading to sustained transcriptional priming of genes such as TNF-α, IL-6, and IL-1β, with IL-1β maturation further enhanced via inflammasome activation (Quintin et al., 2012), (Netea et al., 2016).

In parallel, trained macrophages undergo metabolic rewiring, with a shift toward aerobic glycolysis and tricarboxylic acid cycle remodeling leading to accumulation of succinate and fumarate (Arts et al., 2016a), (Cheng et al., 2014), (Arts et al., 2016b). These metabolites reinforce inflammatory gene expression by modulating epigenetic enzymes and stabilizing hypoxia-inducible factor-1α (HIF-1α), which promotes glycolytic metabolism and supports pro-inflammatory transcriptional programs (Wang et al., 2017). In the chronically inflamed gut, such metabolic adaptations may impair the ability of macrophages to return to tissue-repair phenotypes, thereby promoting mucosal damage. Dysbiosis in IBD further contributes by generating aberrant metabolite profiles that reinforce inflammatory training (Guggeis et al., 2025), (Macias-Ceja et al., 2019), (Fremder et al., 2021), with microbial products including succinate, polyamines, and indole derivatives functioning as metabolic and epigenetic cofactors that directly influence innate immune cell programming (Pålsson-McDermott and O'Neill, 2025).

Importantly, training signals are not confined to the intestine. Inflammatory cues originating from the gut can reach the bone marrow and reprogram hematopoietic stem and progenitor cells, sustaining production of pro-inflammatory myeloid cells that continuously repopulate the intestinal mucosa (Robles-Vera et al., 2025), (Mitroulis et al., 2018). This explains disease persistence and relapse in IBD, even during clinical remission, and suggests that gut-targeted therapies alone may be insufficient if the driving signals originate in the bone marrow.

Notably, the functional consequences of trained immunity are context-dependent. While persistent microbial exposure and barrier dysfunction may promote maladaptive inflammatory training, other forms of innate training may support mucosal repair. For example, β-glucan-induced trained immunity promotes the expansion of reparative Cx3cr1+ macrophages and limits NLRP3 activation through suppression of K+ efflux and mitochondrial ROS generation, facilitating epithelial regeneration in experimental colitis (Lv et al., 2026), (Camilli et al., 2020). Conversely, microbial metabolites such as succinate and polyamines can drive either inflammatory or reparative macrophage responses depending on their concentration and the local inflammatory milieu (Macias-Ceja et al., 2019), (Fremder et al., 2021), (Niechcial et al., 2023), (Li et al., 2025), while butyrate generally favors tolerogenic macrophage programs through inhibition of HDAC3 (Chang et al., 2014), (Eshleman et al., 2024).

Thus, trained immunity should be viewed as a dynamic process capable of driving either mucosal healing or disease progression depending on the context. Rather than broadly suppressing macrophage activity, emerging strategies may seek to selectively modulate trained immune programs, limiting pathogenic inflammation while preserving regenerative functions. Such approaches form the basis of a growing interest in therapeutically targeting macrophage-driven innate immune circuits in IBD.

## Therapeutically targeting macrophage-driven innate immune circuits

Dysregulated macrophage-driven innate immune circuits are now recognized as key drivers of chronic inflammation and defective tissue repair in IBD (Bain and Schridde, 2018). These pathogenic macrophages perpetuate epithelial damage and sustain inflammatory circuits, making them attractive therapeutic targets. Several standard IBD therapies exert indirect effects on macrophages. Anti-TNF-α antibodies and JAK inhibitors attenuate pro-inflammatory signaling, 5-aminosalicylates and corticosteroids modulate transcriptional programs, and granulocyte/monocyte apheresis reduces the recruitment of macrophage precursors into inflamed tissue (Zhao et al., 2026). These findings collectively support the concept of directly targeting macrophages as a therapeutic strategy.

Macrophage-directed therapeutic strategies have demonstrated efficacy in preclinical colitis models by reshaping innate immune programs. One emerging approach seeks to rewire macrophage immunometabolism through manipulation of intracellular NAD^+^ signaling. LMT503 targets the NQO1–NAD^+^ metabolic axis, increasing intracellular NAD^+^ levels and promoting an anti-inflammatory macrophage phenotype characterized by increased expression of SIRT1, SIRT3, and SIRT6, enhanced IL-10 and Arg1 production, and reduced TNF-α and IL-6 secretion, ultimately ameliorating experimental colitis (Lee et al., 2023), (Kim et al., 2025). Regenerative medicine approaches further support this concept: mesenchymal stem cells (MSCs) suppress macrophage glycolysis via the PHD2/HIF-1α axis, limiting IL-1β production and promoting mucosal healing in preclinical colitis models, while MSC-derived exosomes regulate macrophage polarization through the SIRT1–FXR axis (Yuan et al., 2022), (Zhou M. et al., 2025), (Zhu et al., 2023). Early clinical studies with umbilical cord-derived MSCs reported improvements in moderate-to-severe ulcerative colitis (Hu et al., 2016), though the ADMIRE-CD II trial failed to meet its primary endpoint, with remission rates indistinguishable from placebo, raising questions about reproducibility and magnitude of benefit. Ongoing Phase I/II trials evaluating allogeneic bone marrow-derived MSCs (Remestemcel-L) in refractory UC (NCT04543994) and Crohn’s colitis (NCT04548583) may help clarify whether benefits are reproducible and whether efficacy is limited to selected subgroups such as perianal disease.

Immunometabolic interventions further expand the therapeutic landscape. Human studies have demonstrated that CD14^+^ intestinal macrophages from Crohn’s disease patients exhibit enhanced glycolytic activity, which drives inflammatory cytokine production and contributes to disease pathogenesis (Zeng et al., 2024). Notably, TRPM8 signaling regulates calcium-dependent metabolic programming in macrophages, promoting glycolytic activation and inflammatory cytokine production; pharmacological inhibition of TRPM8 using the dietary flavonoid luteolin reprograms macrophage metabolism toward a pro-resolving state, suppresses IL-1β, TNF-α, and IL-6 production, and significantly ameliorates experimental colitis (Cicia et al., 2025).

Inflammasome signaling represents another critical regulatory node in macrophage-driven innate immunity. The NLRP3 inflammasome integrates microbial, inflammatory and metabolic cues to regulate IL-1β and IL-18 secretion, thereby influencing both tissue injury and repair. Experimental studies demonstrate that modulation of NLRP3 activity alters disease severity in murine colitis models (Chen et al., 2024). Importantly, inflammasome activation is tightly coupled to macrophage immunometabolism: enhanced glycolysis and succinate accumulation amplify NLRP3 signaling and inflammatory cytokine production (Fremder et al., 2021), (Littlewood-Evans et al., 2016). Therapeutic interventions targeting upstream metabolic regulators have shown promise: miR-31-5p inhibition activates AMPK/SIRT1, suppresses NLRP3, promotes reparative macrophage repolarization, and attenuates dextran sulfate sodium (DSS)-induced colitis (Yuan et al., 2024). Similarly, Atox1 deficiency reduces ROS-dependent NLRP3 activation, supporting inflammasome–metabolic coupling as a therapeutic strategy (Chen et al., 2024). Direct inflammasome targeting has already entered clinical evaluation in IBD: a phase 1b trial (ISRCTN16847938) of the selective NLRP3 inhibitor selnoflast (RO7486967) in patients with moderate-to-severe ulcerative colitis demonstrated target engagement, suppression of IL-1β signaling, and acceptable safety, although clinically meaningful efficacy was not observed during short-term treatment (Klughammer et al., 2023).

Trained immunity further expands therapeutic opportunities. Recent evidence suggests that β-glucan–induced innate immune training reshapes monocyte differentiation trajectories, expands reparative macrophage populations, and confers protection against DSS-induced colitis through reprogramming of myeloid progenitors (Lv et al., 2026). Although these findings remain preclinical, they highlight trained immunity as a potentially modifiable innate circuit and suggest that redirecting macrophage memory toward pro-resolving programs may offer durable therapeutic benefit.

These studies position macrophage reprogramming as a promising framework for next-generation IBD therapies, with MSC-based approaches and direct NLRP3 inhibition already advancing toward clinical evaluation.

## Conclusion

Macrophage-driven inflammation represents a central pathogenic axis in IBD, integrating epithelial barrier dysfunction, microbial dysbiosis, metabolic rewiring, inflammasome activation and innate immune memory (Scalavino et al., 2024), (Arts et al., 2018), (Saeed et al., 2014), (Zigmond et al., 2014). Beyond simplified polarization models, intestinal macrophages exhibit dynamic plasticity that determines whether inflammation resolves or progresses toward chronicity (Chen et al., 2025), (Garrido-Trigo et al., 2023), (Zhen and Zhang, 2019). Future strategies may benefit from multi-targeted approaches simultaneously addressing macrophage recruitment, immunometabolic programming and inflammasome signaling (Scalavino et al., 2024), (Lu et al., 2024), (Zeng et al., 2024), (Cicia et al., 2025). Disease-specific strategies should further account for the distinct immunopathological features of UC and CD, including the macrophage-intrinsic autophagy defects and transmural fibrotic remodeling more prevalent in CD. Integrating spatial multi-omics with mechanistic and therapeutic insights into a unified framework linking macrophage heterogeneity, metabolic programming and innate immune memory to disease-specific outcomes will be key to advancing precision myeloid-targeted therapies in IBD.
